# English Hospitals on the Continent

**Published:** 1888-03-31

**Authors:** Nurse Edith

**Affiliations:** Fulham


### ENGLISH HOSPITALS ON THE CONTINENT.

Would the Editor or any kind reader tell a certificated nurse the names of any English hospitals on the Continent, France preferred? Nurse Edith.

Fulham.

